# Supplementary material for: The Trajectory of Nutritional Status and Physical Activity before and after Transcatheter Aortic Valve Implantation
Source: Nutrients. 2022 Dec 2;14(23):5137. doi: 10.3390/nu14235137 (PMC9740426; doi:10.3390/nu14235137)
Supplement: Supplementary file 1 [file nutrients-14-05137-s001.zip › nutrients-2014167-supplementary.pdf]

Table S1. complete case analysis of nutritional status and physical activity preprocedural, at 30 days and 6 months after the procedure.

Variable	Preprocedural	30 days	6 months	Adjusted Mixed Linear Model of Change Per Month		
				β	95% CI	*P*
Nutritional status						
MNA-SF score, median (IQR)	13 (12 – 14)	13 (12 – 14)	13 (12 – 14)	0.00	-0.05 , 0.06	0.87
Risk at malnutrition, %	23	23	20	-0.39	-3.27, 2.48	0.79
Physical activity						
Steps per day , mean ± SD	6337 ± 3004	6732 ± 3034	6832 ± 3454	47	-32, 125	0.24
Low physical activity, %	72	53	55	-0.10	-0.23, 0.02	0.11
Peak 30 min cadence, mean	66 ± 19	70 ± 22	69 ± 23	0.22	-0.36, 0.79	0.45
Low intensity (<70 steps/min), %	64	51	53	-0.05	-0.18, 0.08	0.43

Table S2. worst case analysis of nutritional status and physical activity preprocedural, at 30 days and 6 months after the procedure.

Variable	Preprocedural	30 days	6 months	Adjusted Mixed Linear Model of Change Per Month		
				B	95% CI	*P*
Nutritional status						
MNA-SF score, median (IQR)	13 (11 – 14)	13 (11 – 14)	13 (11 – 14)	0.02	-0.03, 0.07	0.50
Risk at malnutrition, %	27	34	35	-0.81	-3.38, 1.75	0.53
Physical activity						
Steps per day, mean ± SD	6273 ± 3007	5723 ± 3603	5756 ± 3650	16	-47, 79	0.62
Low physical activity, %	69	62	62	-0.07	-0.18, 0.03	0.16
Peak 30 min cadence, mean	66 ± 20	61 ± 28	62 ± 27	0.02	-0.41, 0.45	0.94
Low intensity (<70 steps/min), %	61	59	60	-0.01	-0.11, 0.09	0.81

For worst case scenario missing values due to mortality, low health status or inability to contact were imputed with the lowest score of that particular timepoint or were categorized as risk at malnutrition, low physical activity level or low intensity.

Table S3. Change in individual components of the mini nutritional assessment short form.

Variable	Preprocedural	30 days	6 months	A mixed Linear Model of Change Per Month		
				β	95% CI	*P*
Nutritional status						
Lower appetite, %	22	17	19	-0.01	-0.09, 0.08	0.86
Weight loss, %	29	38	32	0.02	-0.05, 0.09	0.64
Poor mobility, %	5	5	6	0.04	-0.11, 0.18	0.62
Stress or sickness, %	11	6	9	-0.01	-0.13, 0.11	0.91
Neurological problems, %	11	12	13	0.00	-0.12, 0.12	0.95
Low BMI, %	20	20	20	-0.02	-0.31, 0.27	0.89
